# Heterogeneous Effects of Calorie Restriction on In Vivo Glucose Uptake and Insulin Signaling of Individual Rat Skeletal Muscles

**DOI:** 10.1371/journal.pone.0065118

**Published:** 2013-06-03

**Authors:** Naveen Sharma, Donel A. Sequea, Carlos M. Castorena, Edward B. Arias, Nathan R. Qi, Gregory D. Cartee

**Affiliations:** 1 Muscle Biology Laboratory, School of Kinesiology, University of Michigan, Ann Arbor, Michigan, United States of America; 2 Department of Molecular and Integrative Physiology, University of Michigan, Ann Arbor, Michigan, United States of America; 3 Department of Internal Medicine, University of Michigan, Ann Arbor, Michigan, United States of America; 4 Institute of Gerontology, University of Michigan, Ann Arbor, Michigan, United States of America; University of Pecs Medical School, Hungary

## Abstract

Calorie restriction (CR) (consuming ∼60% of ad libitum, AL, intake) improves whole body insulin sensitivity and enhances insulin-stimulated glucose uptake by isolated skeletal muscles. However, little is known about CR-effects on in vivo glucose uptake and insulin signaling in muscle. Accordingly, 9-month-old male AL and CR (initiated when 3-months-old) Fischer 344xBrown Norway rats were studied using a euglycemic-hyperinsulinemic clamp with plasma insulin elevated to a similar level (∼140 µU/ml) in each diet group. Glucose uptake (assessed by infusion of [^14^C]-2-deoxyglucose, 2-DG), phosphorylation of key insulin signaling proteins (insulin receptor, Akt and Akt substrate of 160kDa, AS160), abundance of GLUT4 and hexokinase proteins, and muscle fiber type composition (myosin heavy chain, MHC, isoform percentages) were determined in four predominantly fast-twitch (epitrochlearis, gastrocnemius, tibialis anterior, plantaris) and two predominantly slow-twitch (soleus, adductor longus) muscles. CR did not result in greater GLUT4 or hexokinase abundance in any of the muscles, and there were no significant diet-related effects on percentages of MHC isoforms. Glucose infusion was greater for CR versus AL rats (P<0.05) concomitant with significantly (P<0.05) elevated 2-DG uptake in 3 of the 4 fast-twitch muscles (epitrochlearis, gastrocnemius, tibialis anterior), without a significant diet-effect on 2-DG uptake by the plantaris or either slow-twitch muscle. Each of the muscles with a CR-related increase in 2-DG uptake was also characterized by significant (P<0.05) increases in phosphorylation of both Akt and AS160. Among the 3 muscles without a CR-related increase in glucose uptake, only the soleus had significant (P<0.05) CR-related increases in Akt and AS160 phosphorylation. The current data revealed that CR leads to greater whole body glucose disposal in part attributable to elevated in vivo insulin-stimulated glucose uptake by fast-twitch muscles. The results also demonstrated that CR does not uniformly enhance either insulin signaling or insulin-stimulated glucose uptake in all muscles in vivo.

## Introduction

Calorie restriction (CR) without malnutrition (consuming ∼60–75% of ad libitum, AL, intake) has been demonstrated to improve whole body insulin sensitivity in various species, including humans [1], [2], [3], non-human primates [4], dogs [5], rats [6], [7], and mice [8]. Because up to 80% of insulin-stimulated blood glucose clearance is taken up by skeletal muscle [9], it is reasonable to expect that CR leads to increased insulin-mediated glucose uptake in skeletal muscle. Supporting this idea, a number of studies using isolated mouse skeletal muscle [10], [11] or isolated [12], [13], [14], [15], [16], [17], [18], [19], [20], [21], [22] or perfused rat [23] skeletal muscle have reported increased insulin-stimulated glucose uptake for CR versus AL animals.

The mechanisms that account for improved insulin-stimulated glucose uptake with CR have been frequently studied in skeletal muscle under ex vivo conditions. These studies have documented that when skeletal muscle is exposed to insulin ex vivo, CR can enhance the effect of insulin on selected proteins in the insulin signaling pathway that controls the subcellular distribution of GLUT4, the insulin-regulated glucose transporter protein. Phosphorylation of Akt (also known as protein kinase B) on Thr308 and Ser473 is essential for the full effect of insulin on glucose transport [10]. In this context, it is notable that the most consistent result of a series of studies on isolated muscles from CR versus AL rodents has been elevated insulin-induced phosphorylation of Akt on both sites [10], [12], [13], [22], [24], [25], [26]. Akt substrate of 160 kDa (also known as AS160 or TBC1D4) is the most distal substrate of Akt that has been clearly linked to insulin’s activation of GLUT4 translocation [27], [28], [29], [30], [31], [32]. AS160 undergoes Akt-dependent phosphorylation on several sites, with Thr642 and Ser588 being the sites that appear to account for most of insulin’s effect on GLUT4 translocation and glucose transport [28]. CR by 9 mo-old rats was recently reported to lead to greater insulin-mediated AS160 phosphorylation on both Thr642 and Ser588 in isolated epitrochlearis muscle (composed predominantly of fast-twitch, type II, fibers), but not in isolated soleus muscle (composed predominantly of slow-twitch, type I, fibers) despite greater insulin-stimulated Akt phosphorylation in both muscles [22]. These results suggested that the mechanisms accounting for improved insulin-stimulated glucose uptake may not be entirely identical for skeletal muscles with differing fiber type profiles.

In contrast to the numerous studies of CR effects on glucose uptake by isolated skeletal muscles, apparently only one publication has evaluated the effects of several months of CR on insulin-stimulated glucose uptake by individual skeletal muscles under in vivo conditions [7]. Despite having higher whole-body glucose uptake during a euglycemic-hyperinsulinemic clamp, the results of this study surprisingly indicated that CR by 8 mo-old rats (restricted to ∼80% of AL intake beginning at 5 mo-old) did not significantly increase insulin-stimulated glucose uptake by the skeletal muscles that were evaluated [7]. Apparently no previously published studies have reported the effects of CR on insulin signaling by skeletal muscle in response to physiologic insulin concentrations.

The current research was performed to extend the very limited knowledge available about CR effects on insulin signaling and glucose uptake by skeletal muscle under in vivo conditions. The primary goal of this study was to compare adult (9 mo-old) CR (60–65% of AL intake initiated at 3 mo-old) versus age-matched AL rats with regard to glucose uptake and key insulin signaling steps in vivo in multiple muscles stimulated by a physiologic insulin concentration during a euglycemic-hyperinsulinemic clamp. We studied four muscles predominantly composed of fast-twitch (type II) fibers (epitrochlearis, gastrocnemius, tibialis anterior, and plantaris), and two muscles predominantly composed of slow-twitch (type I) fibers (adductor longus and soleus). Because it seemed possible that CR might induce a shift in fiber type composition, we assessed the effect of CR on fiber-type composition in each of these muscles. We hypothesized that CR would influence insulin signaling and glucose uptake in multiple muscles, but the specific CR effects on insulin signaling would not be identical for muscles with different fiber-type compositions.

## Methods

### Materials

Unless otherwise noted, all chemicals were purchased from Fisher Scientific (Hanover Park, IL) or Sigma-Aldrich (St. Louis, MO). Reagents and apparatus for SDS-PAGE and immunoblotting were obtained from Bio-Rad Laboratories (Hercules, CA). Anti-phospho-AS160 Ser588 (pAS160^Ser588^; #3028P2) and anti-phospho-AS160 Thr642 (pAS160^Thr642^; #3028P1) were from Symansis (Timaru, New Zealand). Anti-phospho-Akt Thr308 (pAkt^Thr308^; #9275), anti-phospho-Akt Ser473 (pAkt^Ser473^; #9272), anti-hexokinase II (#2867), and anti-rabbit IgG-horseradish peroxidase conjugate (#7074) were from Cell Signaling Technology (Danvers, MA). Anti-phospho-IR Tyr1162/1163 (pIR^Tyr1162/1163^; #44–504G) was from Invitrogen (Camarillo, CA). Anti-sheep IgG horseradish peroxidase conjugate (#12–342) was from Millipore (Billerica, MA). Anti-GLUT4 was provided by Dr. Samuel Cushman (NIH, Bethesda, MD). West Dura Extended Duration Substrate (#34075) and the bicinchoninic acid protein assay kit (#23227) were from Thermo Scientific (Rockford, IL).

### Animal Care

Procedures for animal care were approved by the University of Michigan Committee on Use and Care of Animals. Male Fischer 344×Brown Norway, F1 generation rats were obtained at 3 months of age from Harlan (Indianapolis, IN). Animals were housed individually in shoebox cages and maintained on a 12∶12-h light-dark cycle (lights out at 17∶00) in specific pathogen-free conditions. The feeding protocol for both the ad libitum (AL) group and the calorie restricted (CR) group has been previously described [22], but briefly the CR group was restricted to 60–65% of AL intake gradually over 3 weeks (90%, 75%, 60–65%). Subsequently, the CR group continued to receive 60–65% of the intake of the AL group daily for ∼6 months.

### Euglycemic-Hyperinsulinemic Clamp

After ∼6 months at the University of Michigan facility, AL and CR rats had catheters surgically placed into the jugular vein and carotid artery one week prior to a euglycemic-hyperinsulinemic clamp experiment as previously described [33]. At approximately 0800 on the day of the clamp experiment, food was removed from rat cages (∼5 h prior to the start of the clamp procedure). The clamp protocol consisted of a 120 min experimental period (t = 0 to 120 min). At t = −10 min, an arterial blood sample (∼100 µl) was taken for assessment of basal levels of insulin and glucose. The insulin infusion was begun at t = 0 with a primed-continuous infusion of insulin (Novo Nordisk, Princeton, NJ). A stable plasma glucose concentration (120–130 mg/dL) was maintained during the clamp by measuring blood glucose every 10 min starting at t = 0 and infusing 50% glucose solution at variable rates accordingly. Plasma insulin concentrations were determined from samples taken at t = −10 and 120 min. Insulin infusion rates were selected with the goal of achieving similar plasma values for insulin in the CR group compared to the AL group. CR rats have a higher rate of insulin clearance as indicated by the elevated C-peptide:insulin ratio in CR compared to AL rats [22] and by the greater insulin binding in the liver of CR versus AL rats after radiolabelled insulin injection [34]. Therefore, to achieve similar plasma insulin concentrations in CR and AL rats, insulin was infused at a higher rate for CR rats (4.7 to 6.0 mU·kg^−1^·min^−1^) versus AL rats (4.0 mU·kg^−1^·min^−1^). To estimate glucose uptake in skeletal muscle, a bolus injection of [1-^14^C]-2-deoxyglucose ([^14^C]2DG; PerkinElmer, Waltham, MA) was given at t = 120 min while continuously maintaining the euglycemic-hyperinsulinemic steady-state. Blood samples were taken at 2, 5, 10, 15, and 25 min after the injection for determination of plasma [^14^C]2DG radioactivity. At the end of the experiment, rats were anesthetized with an intravenous infusion of sodium pentobarbital and muscles were rapidly excised and freeze-clamped with aluminum tongs cooled in liquid nitrogen and stored at −80°C until later processing.

### Plasma Analysis

Plasma glucose during clamps was measured using an Accu-Chek glucometer (Roche, Germany). Plasma insulin was measured using rat/mouse insulin ELISA kits (R&D Systems, Minneapolis, MN).

### Skeletal Muscle Glucose Uptake

For glucose uptake analysis, muscle was homogenized in 0.5% perchloric acid and centrifuged at 2000 *g* for 15 min at 4°C. The supernatants were neutralized with potassium hydroxide (5N). An aliquot of the homogenate was quantified by liquid scintillation counting to determine total tissue values (disintegrations per minute, dpm) for the sum of [^14^C]2-deoxyglucose ([^14^C]2DG) and [^14^C]2-deoxyglucose phosphate ([^14^C]2DGP). Another aliquot was deproteinized with zinc sulfate (0.3N) and barium hydroxide (0.3N) to precipitate [^14^C]2-deoxyglucose-6-phosphate ([^14^C]2DG6P) and quantify [^14^C]2DG in the supernatant. The value for the [^14^C]2DG in the supernatant (dpm) was subtracted from the total tissue [^14^C]2DG and [^14^C]2DGP (dpm) to calculate the glucose uptake rate as indicated by the skeletal muscle [^14^C]2DGP accumulation [35].

### Relative Abundance of Myosin Heavy Chain (MHC) Isoforms

For MHC analysis, muscle was homogenized in pre-chilled glass tissue-grinding tubes (Kontes, Vineland, NJ) containing cold Tissue Protein Extraction Reagent (TPER; Thermo Scientific, Rockford, IL; #78510) supplemented with 1 mM EDTA, 1 mM EGTA, 2.5 mM sodium pyrophosphate, 1 mM sodium vanadate (Na_3_VO_4_), 1 mM ß-glycerophosphate, 1 µg/ml leupeptin, and 1 mM phenylmethylsulfonyl fluoride (PMSF). Protein concentrations were determined using the bicinchoninic acid method (Pierce Biotechnology, Rockford, IL; #23225). Laemmli sample buffer (2×) was then added to 5 µg of the whole homogenate prior to heating for 10 min at 90°C. Samples where then loaded on to gels modified from [36] for SDS-PAGE [Final reagent concentrations for separating gel: 6.5% acrylamide-bis (50∶1), 30% glycerol, 210 mM Tris-HCl (pH 7.4), 105 mM glycine, 0.4% SDS, 19% H_2_0, 0.1% ammonium persulfate, 0.05% TEMED]. Samples were run at a constant voltage (50 V) for 1 h at 4°C with continuous mixing by a magnetic stir bar inside the electrophoresis apparatus. After the initial hour, the power supply was switched from constant voltage mode to the constant current mode. The current value observed at the end of the initial hour was subsequently maintained for an additional 21–22 h at 4°C. Gels were then stained with Coomassie Brilliant Blue (#161-0436, Biorad) overnight at room temperature while gently rotating. The gels were then destained for 4–6 h in 20% methanol and 10% acetic acid solution (destained solution was replaced with fresh solution every 30–45 min). MHC bands were quantified using densitometry (AlphaEase FC, Alpha Innotech, San Leandro, CA).

### Immunoblotting

For immunoblotting analysis, muscle was transferred to microfuge tubes and homogenized in ice-cold TPER buffer as stated above (1 ml/muscle) using Qiagen a TissueLyser II (Valencia, CA). Homogenates were then transferred to microfuge tubes, rotated for 1 h at 4°C, and then centrifuged (15,000 *g*) for 15 min (4°C) to remove insoluble material. The immunoblotting procedure has been previously described [33]. Immunoreactive proteins were quantified by densitometry and normalized to the average value of the AL samples on each blot.

### Glucose-6-phosphate (G6P) and Fructose-6-phosphate (F6P) Concentrations

Frozen muscle was homogenized with a glass mortar and pestle in 0.4 ml of cold 3 M perchloric acid and 1 ml of H_2_0, transferred to a microfuge tube then centrifuged (5000 *g*) for 10 min (4°C). The supernatant (1.2 ml) was then neutralized to a pH of 7.0 and brought to a volume of 3.0 ml with H_2_O in a polypropylene tube, then centrifuged (5000 *g*) for 15 min (4°C). The resulting supernatant was used in the determination of G6P and F6P concentrations by a fluorometric procedure as previously described [37]. Briefly, a Model TD-700 Fluorometer (Turner Designs, Sunnyvale, CA) was used to determine the fluorescence values in samples, and the sample concentrations of G6P and F6P were determined based on the standard curves that were prepared by serial dilutions of analytical grade G6P and F6P (the G6P and F6P values for all of the muscle samples tested were within the range of the standard curves).

### Statistical Analyses

Data were analyzed using SigmaPlot, version 11.0 (Systat Software, San Jose, CA). We performed a student’s t-test for each of the direct comparisons between the two diet groups (AL versus CR). Data are presented as means ± SE. A P value <0.05 was accepted as statistically significant.

## Results

### Body Mass and Daily Food Intake

**Figure 1:**
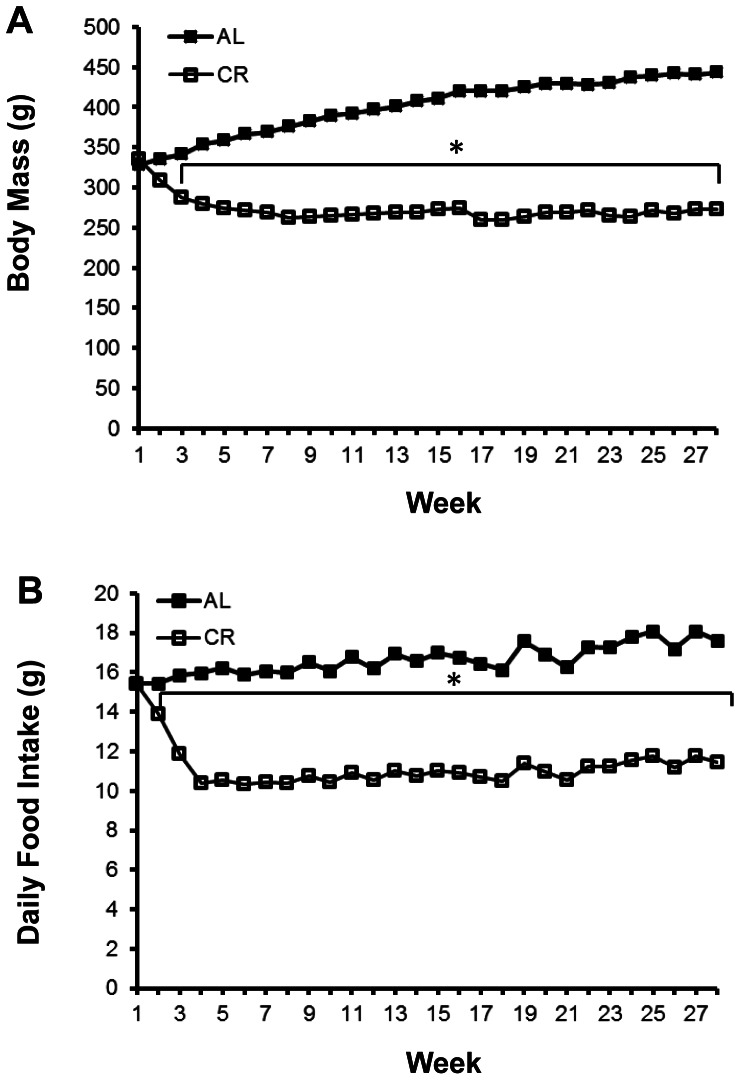
Rat body mass (A) and daily food intake (B). Filled boxes are the AL group and open bars are the CR group. Rats were weighed weekly. Weekly food allotment for the CR group was based on AL group’s food intake for the previous week. When rats were ∼3 mo-old, the food intake of the CR group was reduced to 90% of AL for a week, 75% of AL for the following week, and continued at 60–65% of AL thereafter. *P<0.05 AL versus CR group. Data are means. For clarity, SE bars are not included.

Weekly body mass is shown in Figure 1A. Final body mass of the AL group was significantly (P<0.0001) greater than the CR group (443.36±12.47 g vs. 272.60±7.34 g). As expected, the feeding protocol resulted in the daily food intake of the CR group being progressively reduced during the initial 3 weeks of the intervention, and the CR intake was maintained at 60–65% of the AL group from week 3 onward (Fig. 1B).

### Plasma Glucose, Plasma Insulin, and Glucose Infusion Rate

The baseline plasma glucose concentration was not significantly different between AL (106.5±2.7 mg/dl) and CR (100.9±4.24 mg/dl). Baseline insulin levels were greater (P<0.005) for AL (53.7±5.6 µU/ml) versus CR (28.3±4.5 µU/ml) rats. Glucose levels during the clamp were maintained at 120.7±2.7 mg/dl for AL and 126.1±2.4 mg/dl CR. Insulin levels during the clamp were similar for AL (141.3±9.1 µU/ml) and CR (140.3±6.7 µU/ml). Glucose infusion rates were 78% higher (P<0.001) in CR (31.7±1.6 mg/kg^−1^×min^−1^) versus AL (17.8±0.8 mg/kg^−1^×min^−1^) rats.

### Relative Abundance of Myosin Heavy Chain (MHC) Isoforms

There were no significant diet effects on the percentages of the MHC isoforms (I, IIA, IIX, IIB) in any of the muscles that were studied (Table 1).

**Table 1:** Relative myosin heavy chain (MHC) isoform composition of rat skeletal muscle.

	% MHC I		% MHC IIA		% MHC IIX		% MHC IIB	
	AL	CR	AL	CR	AL	CR	AL	CR
Epitrochlearis	5±2	3±1	8±1	6±1	27±3	24±1	60±4	67±2
Gastrocnemius	13±3	11±2	13±2	12±1	35±3	33±2	39±5	44±3
Tibialis Anterior	3±1	3±1	11±2	11±2	28±1	30±1	58±3	56±3
Plantaris	9±3	10±1	18±2	17±1	41±2	36±2	32±5	37±3
Soleus	99±1	98±1	0±0	1±1	1±1	1±1	0±0	0±0
Adductor Longus	74±6	88±5	8±3	3±1	18±4	9±3	0±0	0±0

Values are means ± SE. MHC isoforms were separated by SDS-PAGE, which were stained with Coomassie Blue. Resulting bands were quantified by densitometry and were expressed as relative values (%) for each of the 6 skeletal muscles.

### 2-Deoxy-D-glucose Uptake

**Figure 2:**
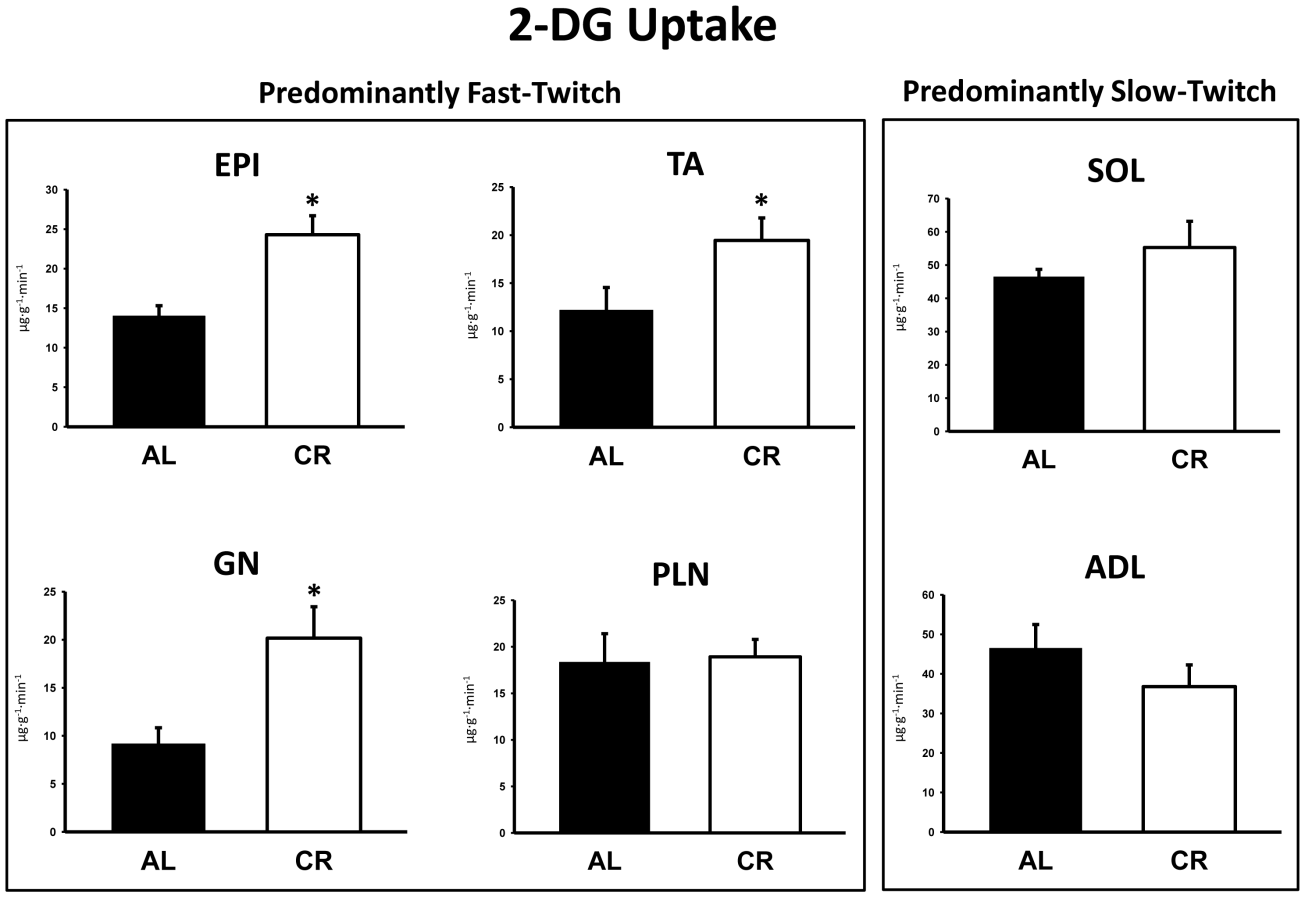
2-Deoxyglucose (2-DG) uptake in predominantly fast-twitch and predominantly slow-twitch muscles. EPI, epitrochlearis; GN, gastrocnemius; TA, tibialis anterior; PLN, plantaris; SOL, soleus; ADL, adductor longus. Filled bars are the AL group and open bars are the CR group. *P<0.05, CR versus AL. Data are means ± SE. n = 6–13 rats per treatment group.

2-DG uptake was significantly (P<0.05) greater in the epitrochlearis, tibialis anterior, and gastrocnemius for CR versus AL rats (Fig. 2). There were was no significant diet effects for 2-DG uptake in the plantaris, soleus, or adductor longus (Fig. 2).

### Immunoblotting

**Figure 3:**
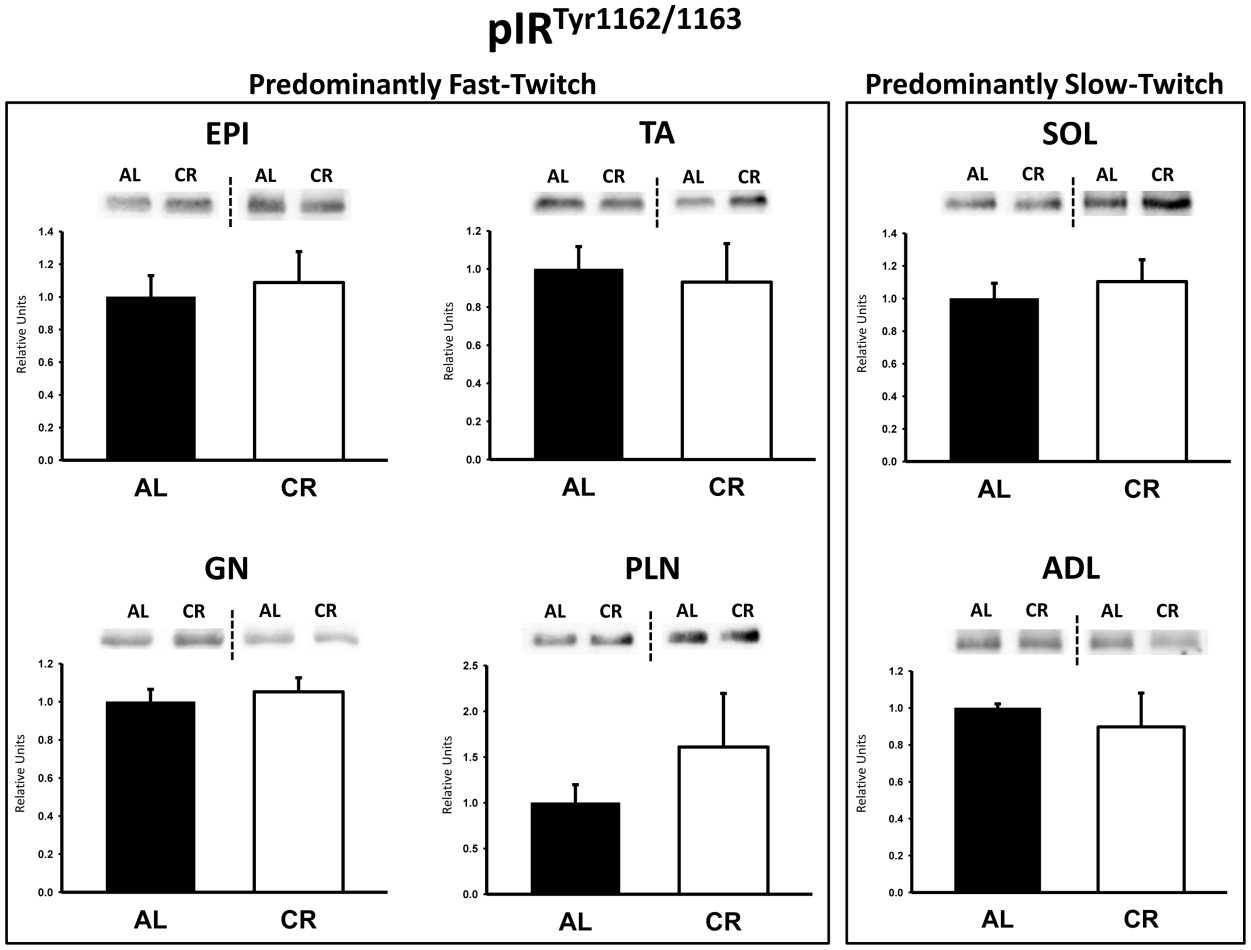
Insulin receptor Tyr1162/1163 phosphorylation (pIR^Tyr1162/1163^) in predominantly fast-twitch and predominantly slow-twitch muscles. EPI, epitrochlearis; GN, gastrocnemius; TA, tibialis anterior; PLN, plantaris; SOL, soleus; ADL, adductor longus. Filled bars are the AL group and open bars are the CR group. Values are normalized to the average value of the AL samples on each blot. Each graph in Figures 3 to 9 is accompanied by representative blots from two muscles from AL and CR rats (the dashed line denotes two independent blots). Data are means ± SE. n = 6–8 rats per treatment group.

**Figure 4:**
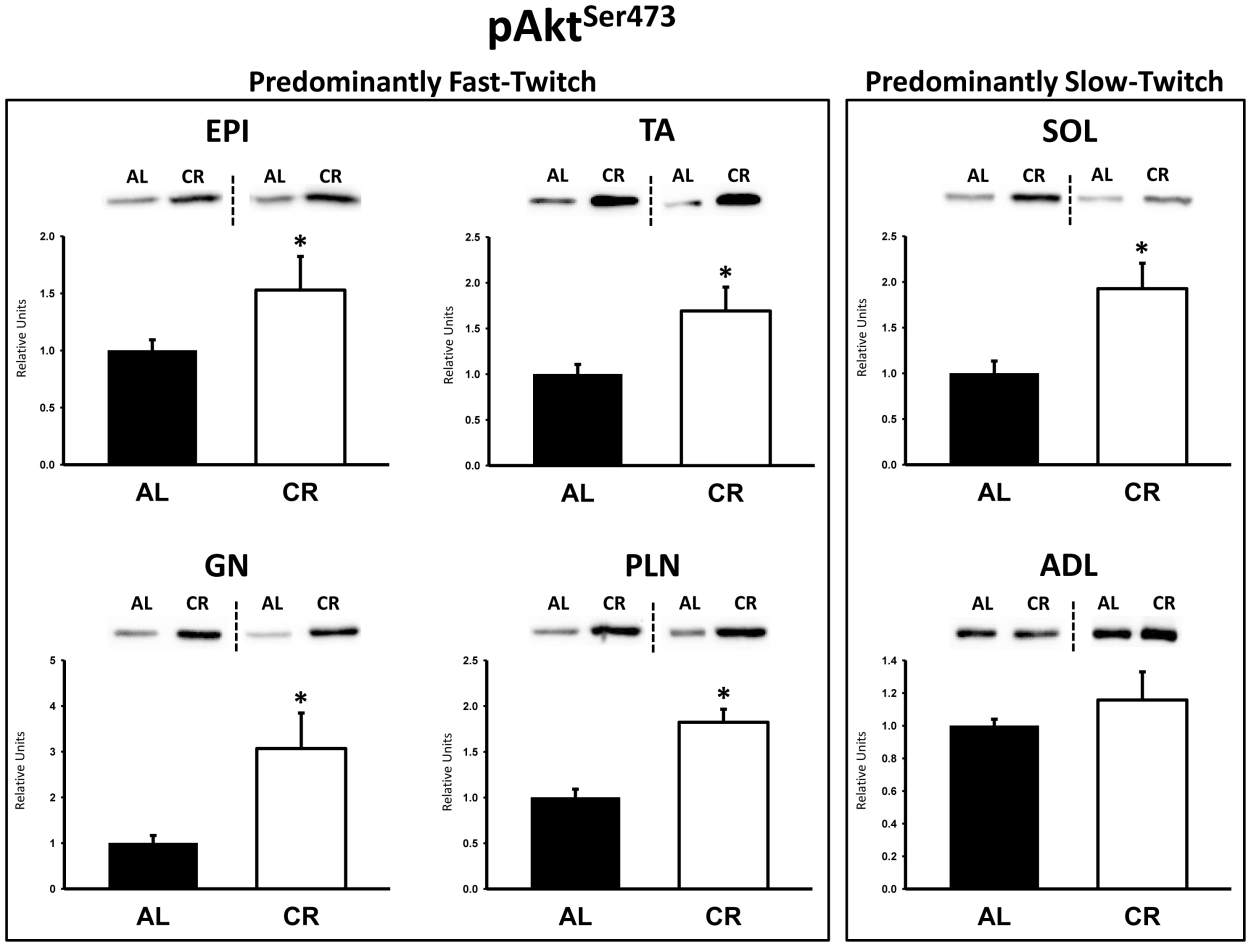
Akt Ser473 phosphorylation (pAkt^Ser473^) in predominantly fast-twitch and predominantly slow-twitch muscles. EPI, epitrochlearis; GN, gastrocnemius; TA, tibialis anterior; PLN, plantaris; SOL, soleus; ADL, adductor longus. Filled bars are the AL group and open bars are the CR group. Values are normalized to the average value of the AL samples on each blot. *P<0.05, CR versus AL. Data are means ± SE. n = 6–8 rats per treatment group.

**Figure 5:**
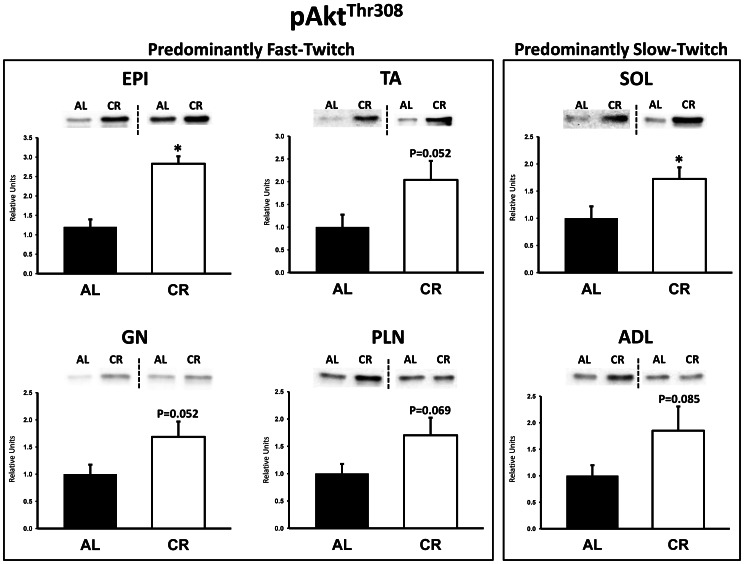
Akt Thr308 phosphorylation (pAkt^Thr308^) in predominantly fast-twitch and predominantly slow-twitch muscles. EPI, epitrochlearis; GN, gastrocnemius; TA, tibialis anterior; PLN, plantaris; SOL, soleus; ADL, adductor longus. Filled bars are the AL group and open bars are the CR group. Values are normalized to the average value of the AL samples on each blot. *P<0.05, CR versus AL. Data are means ± SE. n = 5–8 rats per treatment group.

**Figure 6:**
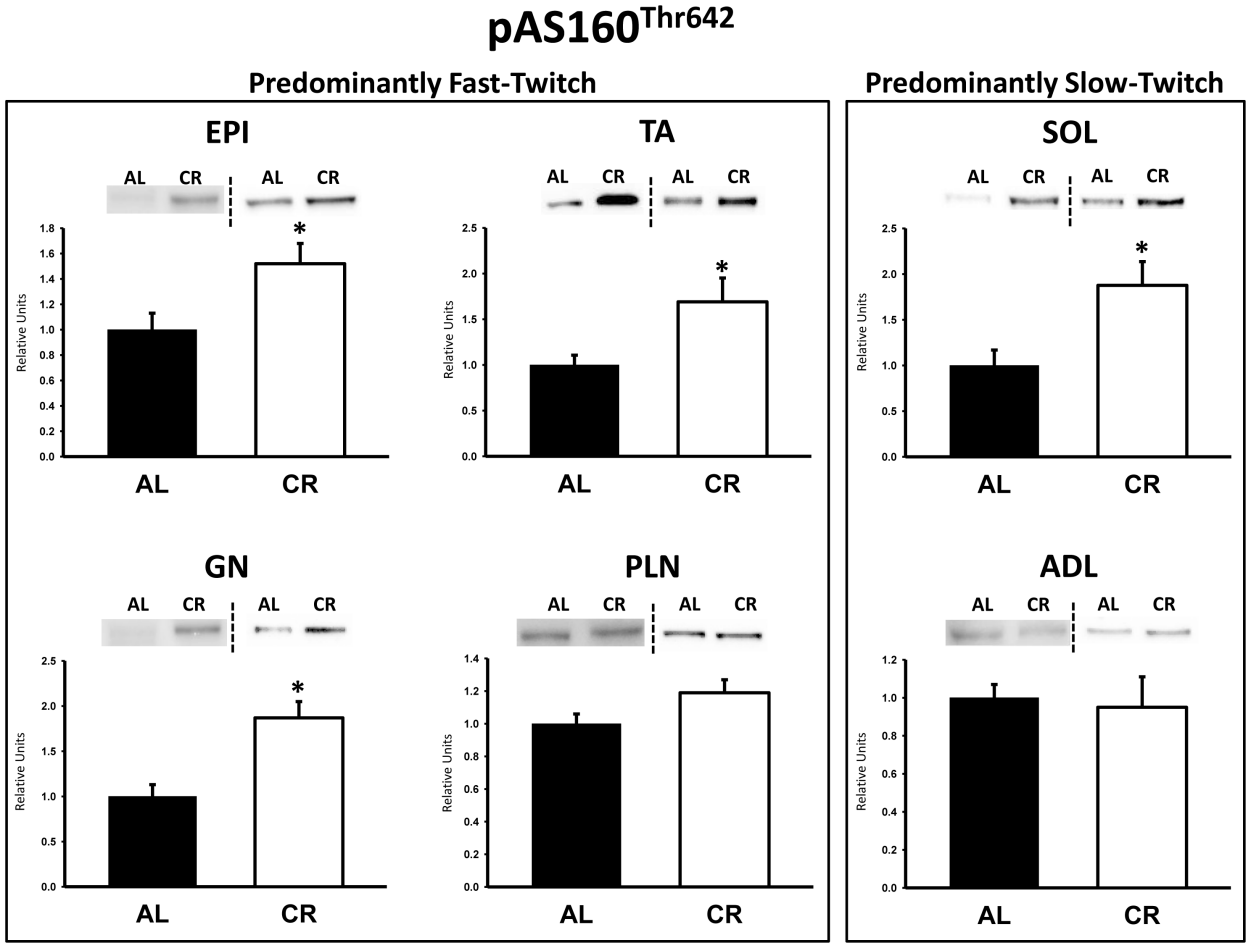
AS160 Thr642 phosphorylation (pAS160^Thr642^) in predominantly fast-twitch and predominantly slow-twitch muscles. EPI, epitrochlearis; GN, gastrocnemius; TA, tibialis anterior; PLN, plantaris; SOL, soleus; ADL, adductor longus. Filled bars are the AL group and open bars are the CR group. Values are normalized to the average value of the AL samples on each blot. *P<0.05, CR versus AL. Data are means ± SE. n = 6–8 rats per treatment group.

**Figure 7:**
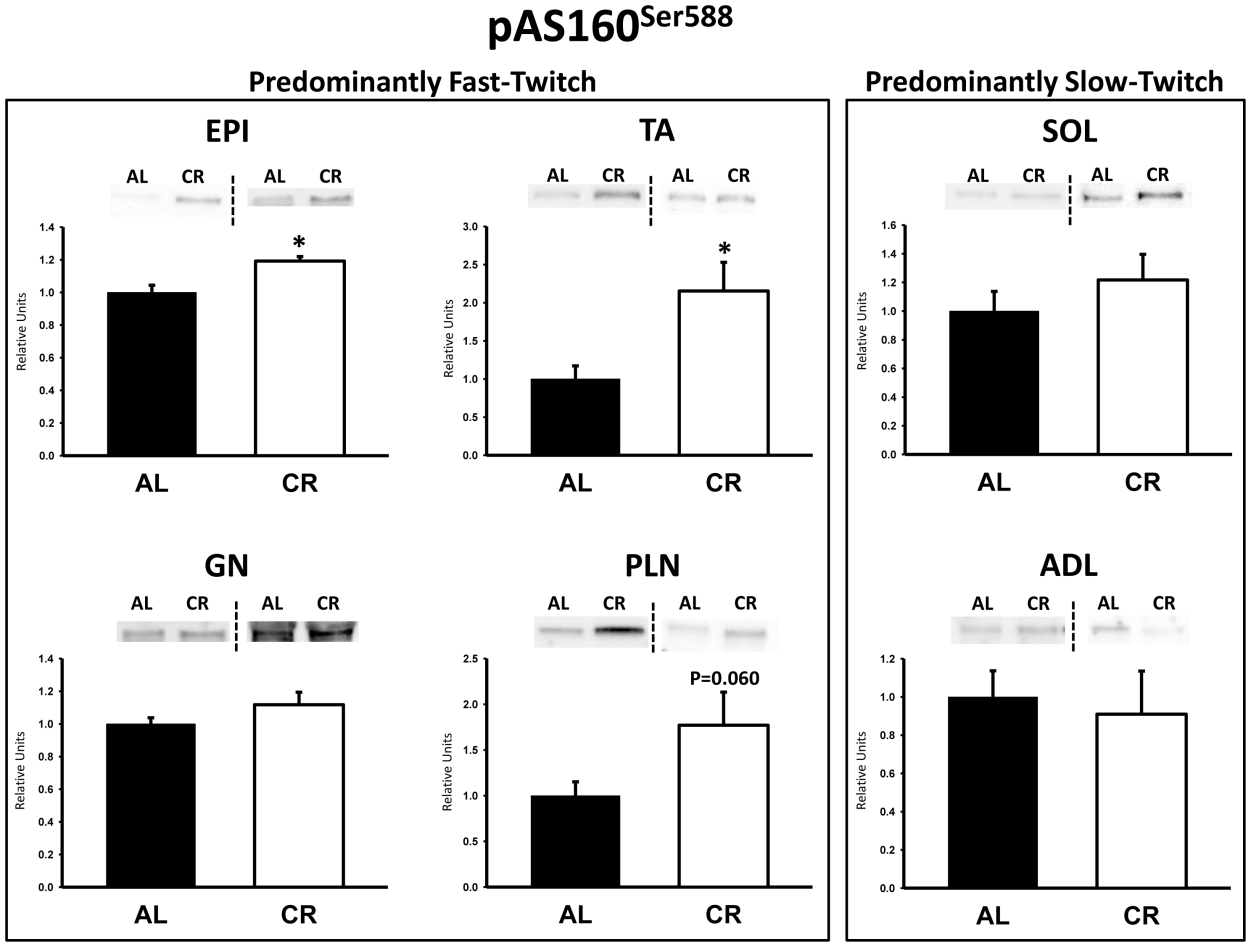
AS160 Ser588 phosphorylation (pAS160^Ser588^) in predominantly fast-twitch and predominantly slow-twitch muscles. EPI, epitrochlearis; GN, gastrocnemius; TA, tibialis anterior; PLN, plantaris; SOL, soleus; ADL, adductor longus. Filled bars are the AL group and open bars are the CR group. Values are normalized to the average value of the AL samples on each blot. *P<0.05, CR versus AL. Data are means ± SE. n = 6–8 rats per treatment group.

**Figure 8:**
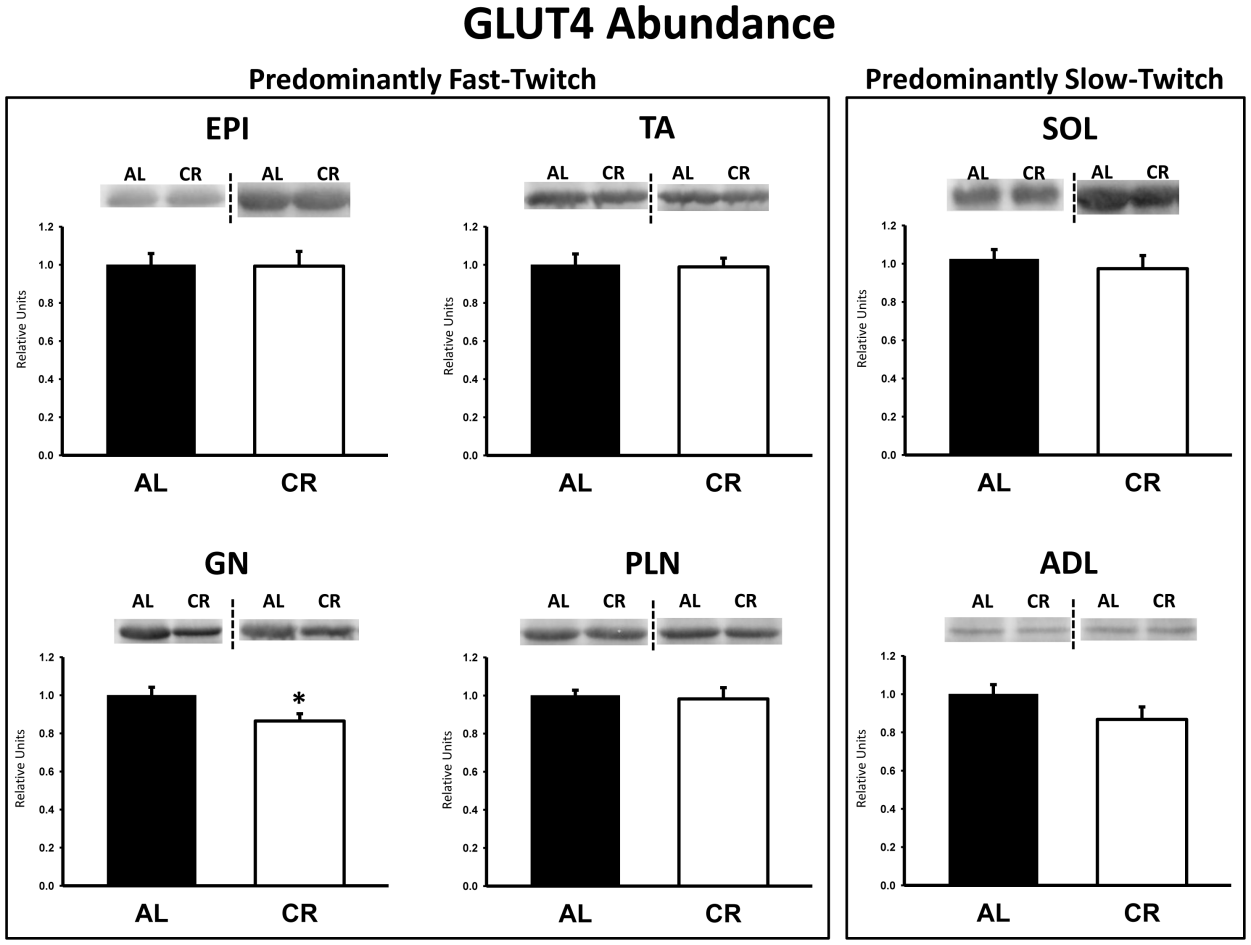
GLUT4 protein abundance in predominantly fast-twitch and predominantly slow-twitch muscles. EPI, epitrochlearis; GN, gastrocnemius; TA, tibialis anterior; PLN, plantaris; SOL, soleus; ADL, adductor longus. Filled bars are the AL group and open bars are the CR group. Values are normalized to the average value of the AL samples on each blot. *P<0.05, CR versus AL. Data are means ± SE. n = 8–13 rats per treatment group.

**Figure 9:**
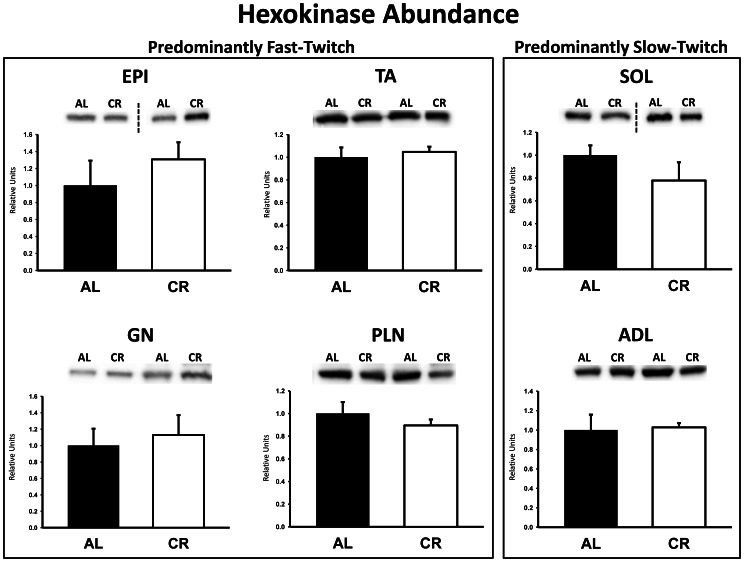
Hexokinase protein abundance in predominantly fast-twitch and predominantly slow-twitch muscles. EPI, epitrochlearis; GN, gastrocnemius; TA, tibialis anterior; PLN, plantaris; SOL, soleus; ADL, adductor longus. Filled bars are the AL group and open bars are the CR group. Values are normalized to the average value of the AL samples on each blot. Data are means ± SE. n = 7–8 rats per treatment group.

There were no significant differences between AL and CR rats on insulin receptor (IR) Tyr1162/1163 phosphorylation (pIR^Tyr1162/1163^) in any of the muscles (Fig. 3). Akt Ser473 phosphorylation (pAkt^Ser473^) was significantly (P<0.05) greater in the epitrochlearis, gastrocnemius, tibialis anterior, plantaris, and soleus for CR versus AL rats, but there was no significant diet effect in the adductor longus (Fig. 4). Akt Thr308 phosphorylation (pAkt^Thr308^) was significantly (P<0.05) greater in the epitrochlearis and soleus, and had non-significant trends in the gastrocnemius (P = 0.052), tibialis anterior (P = 0.052), plantaris (P = 0.069), and adductor longus (P = 0.085) for CR versus AL rats (Fig. 5). AS160 Thr642 phosphorylation (pAS160^Thr642^) was significantly (P<0.05) greater in the epitrochlearis, tibialis anterior, gastrocnemius, and soleus for CR versus AL rats, but there was not a significant diet effect in the plantaris or the adductor longus (Fig. 6). AS160 Ser588 phosphorylation (pAS160^Ser588^) was significantly (P<0.05) greater in the epitrochlearis and the tibialis anterior, and there was a non-significant trend in the plantaris (P = 0.06) for greater values in CR versus AL rats, but there was not a significant diet effect in the gastrocnemius, soleus, or the adductor longus (Fig. 7). GLUT4 protein in the gastrocnemius was slightly, but significantly (P<0.05) lower for CR versus AL rats (Fig. 8). There was no significant difference between AL and CR groups for hexokinase abundance in any of the muscles studied (Fig. 9).

### Muscle Glucose-6-phosphate and Fructose-6-phosphate Concentrations

There were no significant differences between AL and CR groups in either glucose-6-phosphate or fructose-6-phosphate concentrations in any of the muscles studied (Table 2).

**Table 2:** Tissue concentrations of glucose-6-phosphate and fructose-6-phosphate in rat skeletal muscle.

	Glucose-6-Phosphate (µmol/g)		Fructose-6-Phosphate (µmol/g)	
	AL	CR	AL	CR
Epitrochlearis	0.596±0.164	0.759±0.219	0.154±0.038	0.155±0.049
Gastrocnemius	0.119±0.038	0.163±0.038	0.023±0.007	0.037±0.008
Tibialis Anterior	0.366±0.045	0.395±0.073	0.070±0.010	0.083±0.018
Plantaris	0.174±0.031	0.268±0.050	0.046±0.009	0.070±0.009
Soleus	0.106±0.022	0.127±0.029	0.049±0.006	0.054±0.004
Adductor Longus	0.283±0.035	0.278±0.039	0.065±0.012	0.077±0.016

Values are means ± SE. Ad libitum (AL) and calorie restricted (CR) treatment groups, n = 7–8.

## Discussion

CR rats compared to AL rats had greater whole body insulin sensitivity that was, at least in part, attributable to CR effects on glucose uptake by multiple skeletal muscles, including the epitrochlearis, gastrocnemius, and tibialis anterior muscles. Because previous studies have established that improved in vivo insulin sensitivity is a hallmark of CR [1], [2], [3], [4], [6], [7], it was not unexpected that glucose uptake was greater in several insulin-stimulated skeletal muscles of CR versus AL rats during the clamp. However, no CR effect on glucose uptake was found in several other muscles (plantaris, soleus and adductor longus). To gain insights into possible mechanisms for the heterogeneity of CR effects on in vivo muscle glucose uptake, we evaluated muscle fiber type, phosphorylation of several key insulin signaling proteins, GLUT4 and hexokinase protein abundance, and concentrations of muscle metabolites.

Each of the muscles with greater glucose uptake in CR rats was a predominantly fast-twitch muscle, and neither of the predominantly slow-twitch muscles was characterized by a CR-induced increase in glucose uptake. Because insulin-stimulated glucose uptake is higher in muscles enriched with slow-twitch fibers [38], [39], a shift in fast-twitch muscle to a higher percentage of slow-twitch fibers would be predicted to favor greater glucose uptake. However, fiber type composition did not significantly differ between AL and CR rats for any of the six muscles that were studied, so the CR-induced muscle-specific increase in glucose uptake cannot be attributed to altered fiber type composition based on MHC profile. The current observation of increased whole body glucose disposal concomitant with no significant CR effect on soleus glucose uptake in vivo was consistent with the results of Escriva and colleagues [7]. They compared 8 month-old CR rats to age-matched AL rats during a euglycemic-hyperinsulinemic clamp and reported no significant diet effect on soleus glucose uptake along with a greater glucose infusion rate in the CR rats. They also found no significant diet effect for 8 mo-old rats on glucose uptake by either the diaphragm [7] which has a mixed fiber type composition (18% type I, 24% type IIA, 36% type IIB, 22% type IIX) [40] or by the quadriceps which has a fiber type composition (5% type I, 7% type IIA, 66% type IIB, 22% type IIX) [41] that is quite similar to our results for the epitrochlearis. In the current study, we observed no diet effect on glucose uptake by the plantaris which has a fiber type profile very similar to the gastrocnemius. Thus, although available data suggests that CR effects on insulin-stimulated glucose uptake in vivo may be more common in fast-twitch compared to slow-twitch muscles, the muscle heterogeneity for CR-effects cannot be predicted solely on the basis of muscle fiber type composition.

The capacity for insulin-stimulated glucose uptake of skeletal muscle correlates with the muscle’s GLUT4 protein content [38], but GLUT4 abundance was not significantly increased by CR in any of the muscles studied. However, glucose uptake rate depends on translocation of GLUT4 to the cell surface of membranes. We previously reported that the cell surface GLUT4 levels of insulin-stimulated epitrochlearis muscles ex vivo are greater for CR compared to AL rats [15]. In this context, one possibility is that the muscle-specific effects of CR on glucose uptake were attributable to muscle-specific effects on cell surface GLUT4 content. Accordingly, it would be valuable to determine the effect of CR on insulin-stimulated cell surface GLUT4 translocation in multiple skeletal muscles in vivo.

Many previous studies have documented CR-induced increases in insulin-stimulated glucose uptake by isolated skeletal muscles [10], [12], [13], [14], [15], [19], [20], [22], [25], [42], [43]. Especially relevant to the current research is our earlier study that evaluated ex vivo glucose uptake by epitrochlearis muscles [22] from male 9-mo old FBN rats that were either AL or undergoing the same CR protocol as in the current study. Under both in vivo and ex vivo conditions, the epitrochlearis from CR compared to AL rats had enhanced insulin-stimulated glucose uptake concomitant with greater phosphorylation of Akt (Thr308 and Ser473) and AS160 (Thr642 and Ser588). We have previously shown that IR, Akt and AS160 protein levels in the epitrochlearis (as well as other muscles) do not differ for AL compared to CR rats [22], [33]. Using a highly selective Akt inhibitor to eliminate the CR-related increase in Akt phosphorylation, we were able to also eliminate the CR-induced increases in AS160 phosphorylation and glucose uptake by the isolated epitrochlearis [24]. These results provided compelling evidence that CR effects on Akt phosphorylation are essential for the CR effects on glucose uptake, at least under ex vivo conditions. The CR effects on insulin signaling in the epitrochlearis ex vivo correspond closely to the CR effects for insulin signaling in vivo. Therefore it seems reasonable to suspect that the greater in vivo glucose uptake by the epitrochlearis for CR versus AL rats may also rely on greater Akt activation.

Consistent with the results for the epitrochlearis, the other two muscles characterized by greater in vivo glucose uptake for CR rats (gastrocnemius and tibialis anterior) were also accompanied by increased phosphorylation of Akt and AS160 in CR versus AL animals. The CR-induced increases in Akt and AS160 phosphorylation for the gastrocnemius and tibialis anterior may also play a role in the CR-induced increase in insulin-stimulated glucose uptake in these muscles. In contrast, the absence of greater AS160 phosphorylation in the plantaris and adductor longus muscles of CR versus AL rats may be related to the lack of a CR effect on insulin-stimulated glucose uptake in either of these muscles.

The lack of a significant CR effect on in vivo glucose uptake by the soleus in the current study confirmed the observations of Escriva et al. [7]. We extended the earlier results by also demonstrating that in vivo Akt and AS160 phosphorylation of the soleus were greater for CR versus AL rats. Given the CR-related increase in phosphorylation of Akt and AS160 that we found in the insulin-stimulated soleus, what might account for the lack of a concomitant CR-induced increase in insulin-stimulated glucose uptake in this muscle? A novel method that Wasserman and colleagues developed to identify the determinants of in vivo glucose uptake in rat skeletal muscle provides information that is relevant to answering this question [44], [45]. Based on their analysis, they concluded that during a euglycemic-hyperinsulinemic clamp in ad libitum fed rats, glucose transport across the sarcolemma was not the major determinant for glucose uptake by the insulin-stimulated soleus. Instead, they proposed that glucose delivery and/or glucose phosphorylation by hexokinase were the crucial determinants for insulin-stimulated glucose uptake by the soleus in vivo. With regard to these regulatory steps, we did not find CR-induced changes in either hexokinase abundance or G6P concentration, and we are unaware of any information on the possibility that CR alters blood flow to the soleus. Regardless, if in vivo glucose uptake by the soleus of AL rats was not limited by glucose transport, then the CR-induced elevation of Akt and AS160 phosphorylation would not be expected to result in a higher rate of in vivo glucose uptake by the soleus (because these signaling proteins are known to be important for glucose transport, but not for regulating either glucose delivery or glucose phosphorylation). Notably, Wasserman’s group also studied the gastrocnemius and vastus lateralis and reported that, in contrast to the type I soleus, glucose transport was a limiting factor for in vivo glucose uptake by these muscles. Their results in these predominantly type II muscles may be relevant for our observation of substantial CR-induced increases in insulin-stimulated glucose uptake by the gastrocnemius, epitrochlearis and tibialis anterior.

In an earlier study, we found that CR resulted in greater insulin-stimulated glucose uptake in isolated soleus muscles in 9 mo-old FBN rats subjected to the same CR protocol as in the current study [22]. What might account for the differing results for CR effects on glucose uptake by insulin-stimulated soleus ex vivo [22] versus in vivo? The divergent outcomes may be explained by a difference in ex vivo versus in vivo conditions, including: 1) the influence of blood flow (a key determinant of glucose delivery), 2) presence of circulating hormones and cytokines (e.g., adiponectin, resistin, leptin, cortiscosterone, etc.), 3) circulating fuels, 4) neuroregulation, and 5) contractile activity of skeletal muscles. Glucose was absent from the ex vivo incubation media (only 2DG was included), whereas glucose was, of course, present in vivo. The concentration of insulin in vivo (∼140 µU/ml) was ∼30% lower than during the ex vivo condition (200 µU/ml), and the duration of insulin stimulation was almost 3-fold longer during the in vivo clamp (145 minutes) compared to ex vivo incubations (50 minutes). It is possible that a longer exposure to insulin may attenuate the effect of CR on glucose uptake in vivo, although it should be noted that Akt phosphorylation was greater in the soleus of CR versus AL rats at the end of the clamp. To reconcile the results obtained using the different models, it would be valuable to assess in vivo glucose uptake with only 50 minutes of elevated insulin. It would also be useful to perform an experiment in which soleus muscles from AL and CR rats were studied ex vivo with the glucose and insulin concentrations and duration of elevated insulin matched to the values for the clamp procedure.

In conclusion, the current data revealed that CR leads to greater whole body glucose disposal during a euglycemic-hyperinsulinemic clamp that is in part attributable to substantially elevated in vivo glucose uptake by multiple predominantly fast-twitch skeletal muscles. The results also clearly demonstrated that CR does not uniformly enhance either insulin signaling or glucose uptake in all skeletal muscles under these in vivo conditions. The observations in this study provide novel support for the idea that CR effects on in vivo glucose uptake may be, at least in some predominantly fast-twitch muscles, related to enhancing Akt and AS160 phosphorylation. However, the results for the soleus revealed that greater Akt and AS160 phosphorylation are not sufficient for enhanced in vivo glucose uptake by all muscles. It would be valuable to determine the influence of CR on GLUT4 translocation of multiple skeletal muscles when stimulated by insulin in vivo. It would also be useful to perform experiments that identify the underlying causes that account for CR’s different effects on glucose uptake by the soleus under ex vivo versus in vivo conditions. Understanding CR effects under in vivo conditions is our ultimate goal, but the thoughtful use of multiple approaches offers the best opportunity to elucidate the complex biological consequences of CR.
